# Treatment of snoring disorder with a non-ablactive Er:YAG laser dual mode protocol. An interventional study 

**DOI:** 10.4317/jced.56953

**Published:** 2020-06-01

**Authors:** Luís Monteiro, Ana Macedo, Luis Corte-Real, Filomena Salazar, José-Júlio Pacheco

**Affiliations:** 1 Oral Surgery and Oral Medicine Department, University Institute of Health Sciences, CESPU, Paredes 4585-116, Portugal; 2 Cancer Research Group - IINFACTS, University Institute of Health Sciences, CESPU, Paredes 4585-116, Portugal; 3 Oral Diseases Group - IINFACTS, University Institute of Health Sciences, CESPU, Paredes 4585-116, Portugal; 4 Postgraduation Program of Endodontics, University Institute of Health Sciences, CESPU, Paredes 4585-116, Portugal

## Abstract

**Background:**

Snoring disorder is a common problem among world population. Treatment modalities may involve surgical and non-surgical procedures. As main objective we proposed to evaluate the efficacy of non-ablative Er:YAG laser in the treatment of snoring disorder.

**Material and Methods:**

We performed an interventional study in 30 patients with snoring disorders. Three sessions were performed with Er:YAG laser 2940nm in long pulse mode (2J/cm2) and smooth mode (10-8J/cm2) in oropharynx region. We analyzed the efficacy of this protocol using questionnaires for snoring intensity, snoring related characteristics of quality of life (including the Epworth sleepness scale and OHIP-14), the satisfaction of the patients and existence of adverse effects comparing the results before and after the treatment using Wilcoxon Signed Rank test.

**Results:**

There was a 96.7% satisfaction rate after one month of treatment, and 96.4% after 6 months. A reduction of the severity of snoring from 8±1.9 before the treatment to 1.6±1.1 one month after treatment was observed (*p*<0.001). Decrease in mean values of Epworth sleepness scale (9.97±5.3 to 6.54±4.3) (*p*=0.002), and OHIP-14 score (10.9±6.2 to 5.9±5) (*p*<0.001) were also noted. A significant decrease in the Mallampatti and Friedman classification scores were observed (*p*=0.001 and *p*<0.001, respectively). No anesthesia was required, nor adverse effects were observed.

**Conclusions:**

Non-ablative Er:YAG laser treatment is a safe, painless, and can be an effective treatment option to reduce snoring and is well accepted by the patient. However, further controlled studies with longer follow-up are required.

** Key words:**Er:YAG laser, snoring, sleep disorders, epworth sleepiness scale, OHIP-14.

## Introduction

Snoring is a common social problem worldwide, with studies indicating that 45% of people often snore, more often in man and elderly people (1-3). Snoring sound could result from the vibration of the oropharynx tissues when there is a partial collapse of the upper airways, specially of soft palate, lateral walls of the pharynx, and the base of the tongue (2).

Snoring disorder may be implicated with health problems such as sleep deprivation, headaches, irritability, decreased concentration and sexual libido, social interrelation capacity, and obstructive sleep apnea (OSA). Some studies also indicate a positive relationship between a high snoring and the risk of stroke and even death. Moreover, snoring is considered a problem and often causes marital problems, and may even lead the couple to sleep in separate rooms commonly (1-5).

Several risk factors contribute to this condition such as obesity, alcoholism, consumption of sedatives, smoking, old age, anatomical variations that causes airway obstruction including long soft palate and also uvula with a longer length, hormonal problems, low tonus of the muscles of the throat or tongue, and hypertrophy of tonsils (1-3).

The diagnosis of snoring is based on a detailed clinical history and examination, and using adjuvant exams such as polysomnography (6,7). The presence of patient´s companion or couple member is important, since symptoms such as snoring or the presence of small awakenings are often not noticed by patient himself.

The treatment modalities for this disorder may involve surgical and non-surgical procedures. The first intervention is related with the elimination of alcohol, smoking habits and promotion of exercise and controlled diet (1,3). Then, several options include mandibular advancement devices, or even positive pressure device (CPAP), here in cases of snoring associated to obstructive sleep apnea syndrome (OSAS) (1,3). Surgical and highly invasive procedures such as uvulopalatopharyngoplasty (UPPP) and laser uvulopalatoplasty are sometimes indicated (LAUP) (1-3,7,8). However, most of these procedures have several limitations, such as possible low success rates or unpredicTable results and noncompliance of therapy by patients (6,8-10).

Recently, an non-invasive treatment using Er:YAG laser in a non-ablative mode has been reported with the aim of the remodelling and new formation of collagen fibers leading to shortening of the mucosa and an increase of the oropharynx space facilitating the airflow passage (1-3,11). This could be an alternative to existing treatments for the treatment of snoring and OSAS. However, the impact of this treatment including in several parameters of the quality of life of these patients is scarce. We hypothesize that the treatment of snoring disorder with non-ablative Er:YAG laser protocol is capable of decrease the intensity of snoring without significant complications. In the view of this, our aim is to evaluate the effectiveness of a non-ablative Er:YAG laser protocol in the treatment of snoring disorder evaluating several snoring related characteristics of quality of life, the satisfaction of the patients with the treatment and existence of adverse effects or complications comparing the results before and after the treatment.

## Material and Methods

-Study population

**Figure 1:**
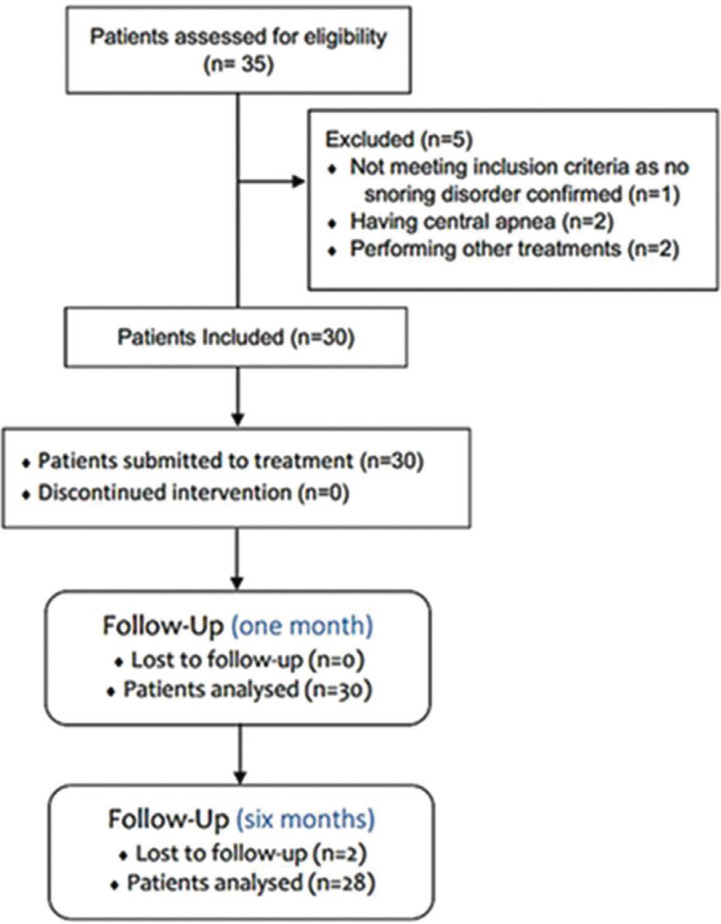
Flow-chart of the included patients. The recruitment and diagnosis of patients started at day 1 of January and stop at the first of June of 2018. The treatment was performed between 1 March to 31 of June and the 31 of July corresponded to last day for eventual one-month follow-up period. Additionally, some variables were evaluated by telephone interview after 6 months of the treatment having 31 of December as last day.

This was an interventional prospective study with a cohort of patients with snoring disorder attending at the University Clinic of the Nova Saúde SA – University Institute of Health Sciences (IUCS), Oporto, Portugal between day 1 of January 2018 to 31 of December of 2018. The study was performed in accordance with the Declaration of Helsinki and after the permission of the institutional ethical board of the University (IUCS Ethical Council) (16-CE-IUCS/2018). Patients attending to the University Clinic of the Nova Saúde SA because snoring were initially observed and then were send to a sleep disorder specialist appointment to perform a complete clinical examination of nose and throat (including video/endoscopy, computed tomography and polysomnography). The inclusion criteria for the study were patients with diagnosis of snoring disorder, either sex, aged 18 year-old or more. Exclusion criteria included patients younger than 18 years, pregnant women, patients with cardiac problems, with diagnosis of central apneas or larynx obstruction, patients taking photosensitive drugs, patients with alcohol habits misuse, patients already surgically treated or with other treatments in course (including oral appliance - MAD), patients who were not able to perform the 3 sessions or follow-up appointments and non-cooperating patients. After applying the inclusion and exclusion criteria, of the initially 35 eligible patients, 5 were excluded (2 patients had performed other treatments before, and 3 were excluded by sleep specialist because two having central apnea and other patient with no confirmation of snoring disorder) (Fig. 1). The final sample was composed by 30 patients, 22 females and 8 males, with a mean age of 41.8±12.8, median age 42 (range 18 to 65 years-old).

-Laser Procedure

The treatment Laser procedure were performed in the Oral Laser Unit of the University Clinic of the Nova Saúde SA (IUCS), Oporto, Portugal. The patients were all treated in three sessions (at 1, 14 and 28 days) with a 2940 nm wavelength Er:YAG laser (Lightwalker AT-S, Fotona®, Slovenia) with a non-contact irradiation of connective and muscles tissue of oropharynx (soft palate including uvula, anterior and posterior pillar´s, and rest of oropharynx) using a PS04 handpiece with 7-mm spot size. The parameters were set as a combination of a long pulse (LP) with a fluence of 2J/cm2 with 12Hz in a brushing technique with 6 passes in a well-defined overlays and a smooth mode with a fluence of 10-8J/cm2 with 2Hz performing 4-6 smooth pulses with 6 passes, with an overlap around 50%, with total pulses ranging between 10,000 and 12,000 pulses per session. Usual safety precautions related with the instrument for protecting the operator, patient, and assistant were followed.

At the end of each session the patient was asked the level of pain (VAS 0-5) and a photograph of the patient’s oral cavity was also taken.

-Outcome measures

Several clinical data including clinical history and physical examination, were recorded before and after the laser procedure in order to evaluate the efficacy of this protocol. During physical examination the patients were classified according to Mallampati anatomical classification (visibility of tonsils, uvula and soft palate), Friedman Tongue classification (FTP) and also according body mass index (BMI) (12,13). Patients whose body mass index was between 25 Kg/m2 and 29.9 Kg/m2 were considered overweight and 30 Kg/m2 or more indicated obesity (13).

Additionally, 3 questionnaires were performed. The snoring general questionnaire according to Miracki & Vizintin (cited in Storchi *et al.*) (2) was carried out and consisting of eight questions, assessing the severity of snoring (the first question) and other problems associated with sleep-disordered breathing (questions 2-8). Answers were graded on 11-point scales (0-10) and two scores were taken as the main outcome measure: the snoring severity alone (Q1) and the total questionnaire (SGQ) score. The opinion of the respective spouses or persons who shared home with the patients were taken into consideration for the evaluation of the patient’s improvement helping the patient in his own perception of snoring. Possible adverse effects in the post-treatment period before and after each treatment session were also recorded. Additional questionnaires were performed including the Epworth Sleepiness Scale (ESS) (ranging 0 to 24 points) (13) and a quality of life for oral health related problems OHIP-14 (15), a questionnaire with 14 questions (ranging 0 to 70 points). We considered the evaluation before the treatments and in a follow-up period after one month the treatments for all outcome variables by clinical examination and also 6 months after treatment for general questionnaire including the satisfaction of the patients (no satisfied or satisfied) by telephone interview.

We also used the mobile phone application Snorelab®, an audio-recording application for patients who snore, in order to obtain a chart recording the patient’s snoring intensity. Patients would have had to perform the application the night before the first treatment and the night after they performed each laser session and then in the follow-up period of one month after.

-Statistical analysis

The data analysis was obtained by descriptive and inferential statistics, using the SPSS-24.0 software (Statistical Package for Social Sciences). The results were expressed in absolute and relative frequencies. Non-parametric tests were used to analyze possible relations between variables (Wilcoxon matched-pairs signed ranks test, and McNemar test). Differences were considered statistically significant at *P*<0.05.

## Results

Of the initial 35 patients observed referring snoring and after applying the inclusion and exclusion criteria, a total of 30 patients were included and evaluated in the study (Fig. 1). Of these, 22 (73.3%) were male and 8 (26.7%) were female. The ages of patients range from 18 years to 65 years-old, with a median age of 42 years-old. The median body mass index (BMI) corresponded to 28.2 Kg/m2 (mean of 27.4±4.7) where 8 (26.7%) patients had BMI less than 25 considered as normal, while 11 (36.7%) were overweight and 11 (36.7%) were obese. Less than 27% of the patients were smokers and 80% had alcoholic habits (only regular drinking without misuse drinking). According to Mallampati classification, 8 (26.7%) patients were class IV, 12 (40%) class III, 9 (30%) class II and only one (3.3%) was class I. Other clinical and demographic variables are reported in Table 1.

**Table 1:**
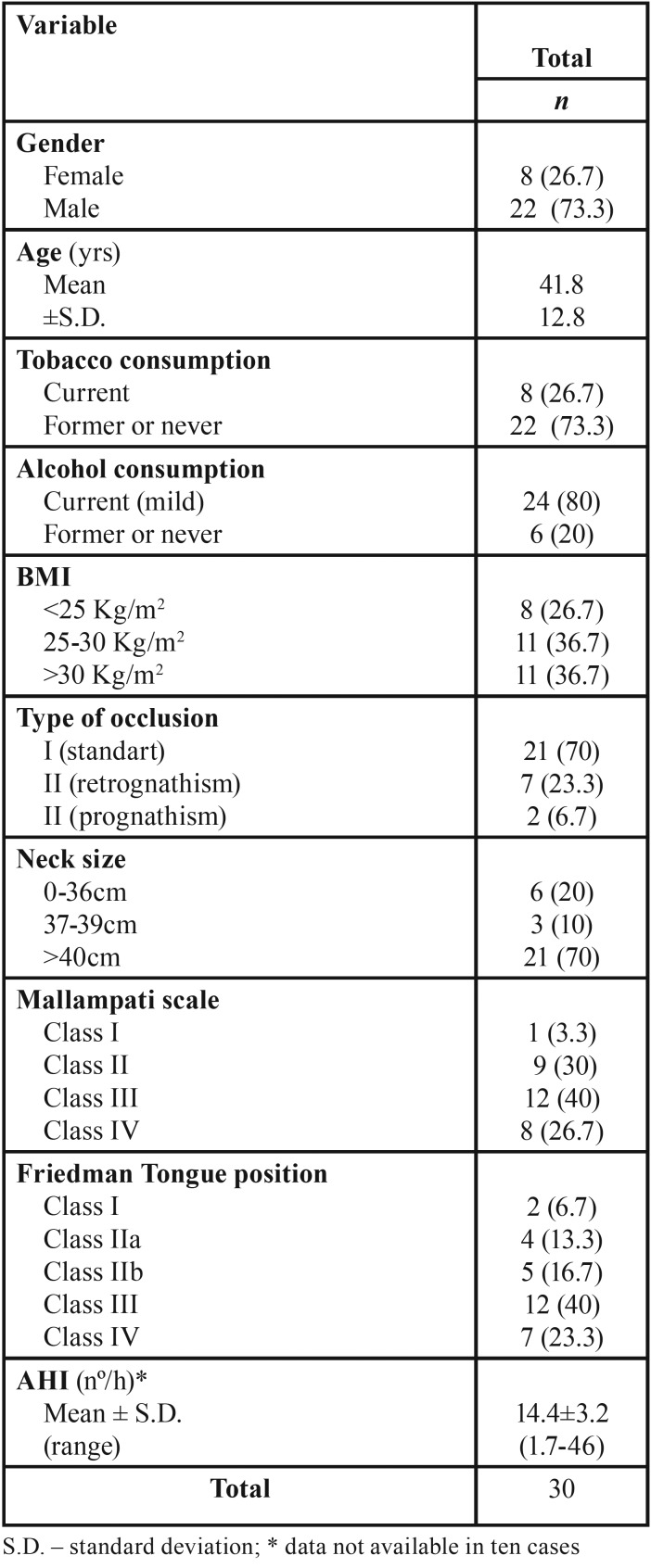
Patient´s characteristics included in the study.

All patients included in the study performed the 3 laser sessions. All patients evaluated the pain/discomfort experienced during treatment (0-5). After the first session the mean pain was 0.47 points (range 0-1), after the second session was 0.37 (range 0-1) and at the last session corresponded to 0.37 (range 0-1). There was no need to use any type of anesthesia in any case. Each session had an approximated duration of 30 minutes.

**Figure 2:**
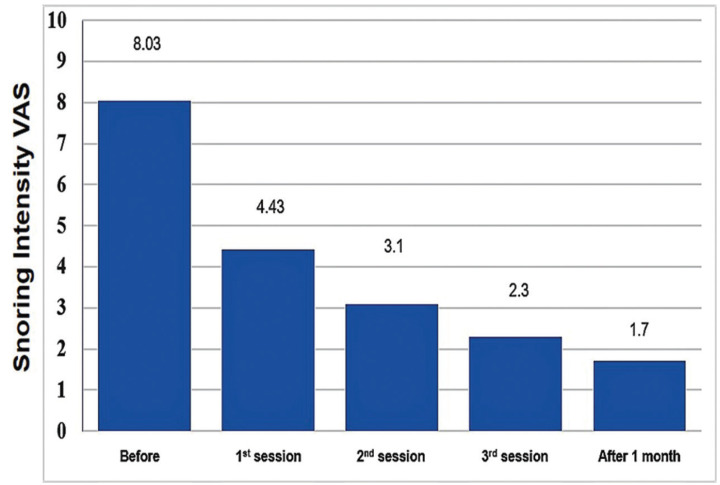
Snoring VAS (snoring intensity own perception) reported before the treatment and after each treatment (TT) session and 1 month after.

In reevaluation after one month, a decrease in the Epworth sleepiness scale, in the snoring intensity VAS as well as in several aspects of the quality of life of the patient was verified (Table 2). The severity of snoring was evaluated through the VAS scale (Table 2; Fig. 2), which showed reduction in the initial mean value of 8.03±1.9 to 4.43±1.9 shortly after the first session and a mean value of 1.7±1.1 after one month of treatment (*p*<0.001), which corresponds to a decrease of 78.8%. Dry mouth sensation upon waking decreased shortly after laser treatment as well as difficulty in waking up in the morning and the sensation of being tired during the day have also been reduced (*p*<0.001). When we use the SGQ questionnaire, again a significant reduction was observed in each session and specially after one month the end of the treatment from the initial value of 26.3±11.2 to a final value of 2.9±2.5 (*p*<0.001) (Table 2), which corresponds to a decrease of 89%. We performed also an oral questionnaire related with oral mouth quality of life and we observed a reduction from an initial value of 10.9±6.2 to a value of 5.9±5 at the end of treatment (*p*<0.001).

**Table 2:**
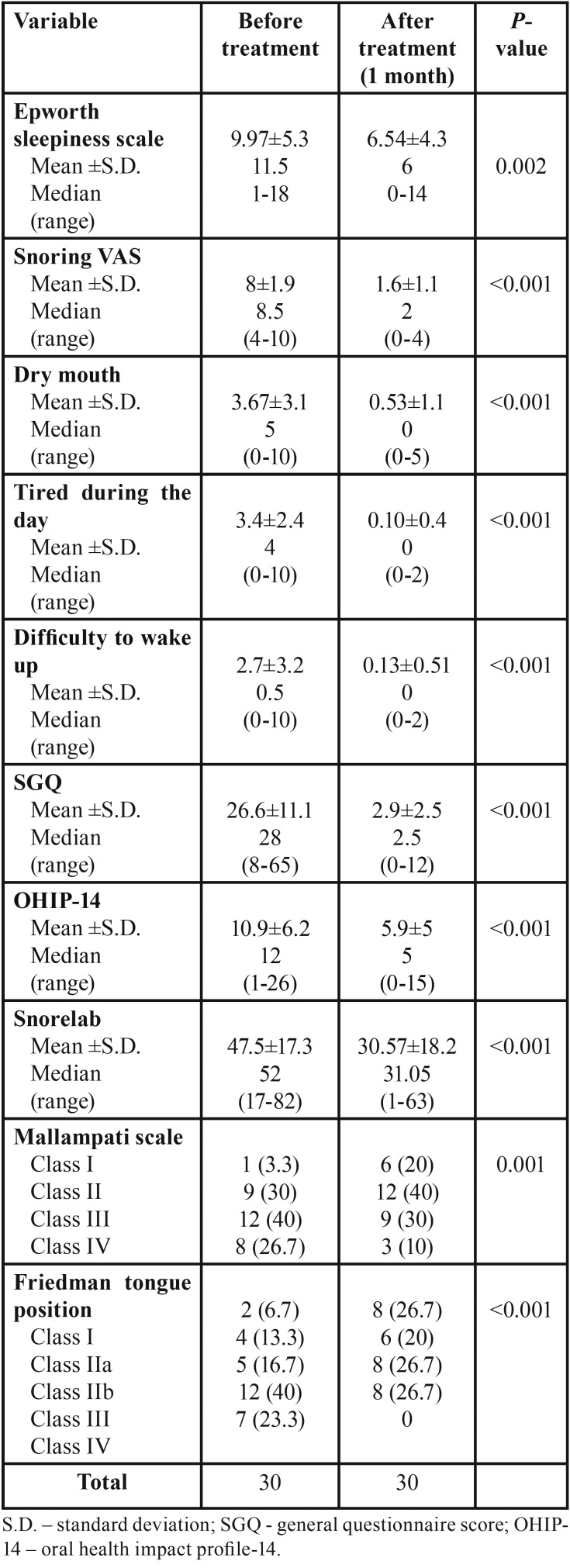
Patient´s characteristics related with snoring before and after the treatment.

With Snorelab® software, a reduction of 22% in the snoring percentage was achieved shortly after the first session (Snorelab® median values of 52% before treatment to 30% after the first treatment) and 20% one month after the end of the 3rd session (Snorelab® values from 52% to 32%) (*p*<0.001).

Regarding the evaluation of anatomical position of tongue and palate thought Mallampati and Friedman tongue position classification we observed a significant decrease in the levels of both scores as we can observe in Table 2.

We also assessed the patient satisfaction of the treatment after one month and 96.7% of the patients were satisfied with the results. At this time 14 (46.7%) patients answered that they maintained a stable result of the effect of the treatment, 1 (3.3%) stated a partial loss of effect and 15 (50%) affirmed an improvement in the result. No patient reported adverse effects at one-month follow-up. Two patients were lost to follow-up at 6 months. The remaining 28 patients were interviewed by telephone and we obtained 96.4% of patient satisfaction, a maintaining or improvement of the effect of the treatment was reported by 92.9% of patients and a mean snoring VAS of 1.9.

We performed a sub-analysis regarding age, BMI, Mallampati and Friedman classification and didn´t find statistical differences in the evaluated outcome variables between these subgroups.

## Discussion

Snoring is a problem with a strong impact on social and specially marital relationships, and can be also an indicator of diseases with risk for the life (1-5). Currently, many treatments can be performed, but many of them are too invasive, with significant adverse effects, with need of general anesthesia and many times with high recurrences rates (7-10). New non-invasive methods for this condition, as main treatment or in combination with other existent options are needed in order to improve the patients’ quality of life. Our aim in this work was to evaluate the effectiveness and safeness of a non-ablative Er:YAG laser protocol in the treatment of snoring disorder.

We observed an improvement of snoring VAS score in 78.8% (8.03 to 1.7) of the patients at one month after the treatments. This result is in line with the other studies with a final overall improvement range of 65% to 85% (2). We observe an improvement of 45% immediately after the first session, 61% after the second session, and 69% after the third session. Storchi *et al.* (2) observed a 70% of VAS score reduction after the three treatments. Additionally, to the improvements in the snoring reduction, we observed an improvement in sleep quality and other quality of life variables. We observed a decreased in the general SGQ questionnaire of 89% after one month of follow-up but also in specific in score variables such as “Tired during the day”, “Difficulty to wake up” and other variables assessed by the SGQ questionnaire. We performed also the OHIP-14 questionnaire (15), commonly used for the evaluation of oral health problems. We observe that patients were feel better of oral health conditions after treatment. This is an important result as oral cavity could be affected by symptoms related with snoring disorders.

We introduce a mobile phone application Snorelab® in order to obtain a chart with the record of the patient’s snoring intensity. We have confirmed a significant reduction of the snoring intensity, shortly after the first session and also at the follow-up of one month. This is an important result as represent a more objective and easily measure of the daily snoring intensity for the patients. Nevertheless, some information about the use of this software must be given to the patients to prevent some bias that could occur during the utilization this software. The same conditions before and after the treatment monitoring must be critically followed. This application could be also important allowing an increase in the awareness of the condition for the patients and could be used in a routine daily base.

When we analyse Mallampati and Friedman classifications at one month after the treatment a significant decrease in scores were noted which could suggest that an increase in oropharynx airspace was obtained for these patients. We acknowledged that these classifications could be subjective and affected by several conditions, but other studies have shown an increase in oropharynx airflow after non-ablative Er:YAG laser procedure in human and lab studies (11,22). Lee and Lee (11) in a pilot study with 7 patients using cone beam computed tomography reported an increase in the mean total airway volume 12 weeks post-laser treatment. This effect could be a result, initially, of shrinking of collagen fibers of treated area and then of the formation of new collagen fibers resulting in a remodeling of oropharynx space (16-21). The shrinkage of collagen tissue was also demonstrated in a histologic study of the soft palate of 20 rats submitted to a non-surgical Er:YAG laser procedure (22).

No relevant complications were present and the treatments were well accepted by the patients. There was no need for anesthesia and all treatments were performed in all patients as reported by others (1). This suggest the safeness of this Er:YAG laser protocol that is in line with other reports (1-3,11).

Almost all patients were satisfied with the obtained results of the treatment and many of them had an improvement in their result at one month of follow-up. This could be related with the collagen remodeling that could be expected in a period of few months after treatment (16,20). Patients were interviewed by telephone after 6 months the end of the treatment and the rate of satisfaction was maintained the same confirming that this is an efficient protocol after even 6 months of the end of last session.

We could not find differences in the results regarding age, gender, BMI, Mallampatti or Friedman classification. Although, this need further investigation with studies with bigger samples and long follow-up time to confirm this, our results suggest that this treatment can be used in several patients regarding age or BMI characteristics. In fact, some of highest reduction in snoring index were observed in obese patients.

We acknowledge some limitations or potential bias of the presented study, such as the limited number of patients included in the sample or the short follow-up time of evaluation. Unfortunately, in some cases, we had a limited access to the complete polysomnography’s data performed by patient´s and specially after treatment as observed in other studies (2). However, all patients included in this study were diagnosed with snoring disorder by specialist in sleep disorders (performing the adjuvant exams necessary for diagnosis) and have given authorization for inclusion in our treatment protocol. Moreover, we have evaluated several variables related to snoring such as Epworth Sleepiness Scale (ESS), Mallampati anatomical classification, Friedman Tongue classification and also a more objective tool, the mobile phone application Snorelab®. To our knowledge this is the first report of a series of selected cases reporting a significant decrease in intensity of snoring confirmed by Snorelab® software with an increase in the quality of life related with oral health measure by the OHIP-14 score.

As conclusion, our results showed that non-ablative Er:YAG laser protocol used here is a safe, painless non-invasive and effective treatment option to reduce snoring and is well accepted by the patient. Interestingly, an improve in the quality of life related with mouth health and snoring characteristics was also demonstrated with results observed shortly after the first treatment. Further studies are required with longer follow-up, and especially with control arm group.
